# Peptide OM-LV20 protects astrocytes against oxidative stress *via* the ‘PAC1R/JNK/TPH1’ axis

**DOI:** 10.1016/j.jbc.2022.102429

**Published:** 2022-08-28

**Authors:** Saige Yin, Ailan Pang, Chengxing Liu, Yilin Li, Naixin Liu, Shanshan Li, Chao Li, Huilin Sun, Zhe Fu, Yinglei Wang, Yue Zhang, Meifeng Yang, Jun Sun, Ying Wang, Xinwang Yang

**Affiliations:** 1 Department of Anatomy and Histology and Embryology, Faculty of Basic Medical Science, Kunming Medical University, Kunming, Yunnan, China; 2 Department of Neurology, First Affiliated Hospital of Kunming Medical University, Kunming, Yunnan, China; 3 Department of Biochemistry and Molecular Biology, Faculty of Basic Medical Science, Kunming Medical University, Kunming, Yunnan, China; 4 Key Laboratory of Chemistry in Ethnic Medicinal Resources & Key Laboratory of Natural Products Synthetic Biology of Ethnic Medicinal Endophytes, State Ethnic Affairs Commission & Ministry of Education, School of Ethnic Medicine, Yunnan Minzu University, Kunming, Yunnan, China

**Keywords:** ischemic stroke, astrocytes, TPH1, peptide OM-LV20, ANS, anisomycin, BCA, bicinchoninic acid assay, CAT, catalase, CNS, central nervous system, FBS, fetal bovine serum, I/R, ischemia-reperfusion, IS, ischemic stroke, RT-qPCR, quantitative real-time PCR, SD, Sprague–Dawley

## Abstract

Stroke can lead to severe nerve injury and debilitation, resulting in considerable social and economic burdens. Due to the high complexity of post-injury repair mechanisms, drugs approved for use in stroke are extremely scarce, and thus, the discovery of new antistroke drugs and targets is critical. Tryptophan hydroxylase 1 (TPH1) is involved in a variety of mental and neurobehavioral processes, but its effects on stroke have not yet been reported. Here, we used primary astrocyte culture, quantitative real-time PCR, double immunofluorescence assay, lentiviral infection, cell viability analysis, Western blotting, and other biochemical experiments to explore the protective mechanism of peptide OM-LV20, which previously exhibited neuroprotective effects in rats after ischemic stroke *via* a mechanism that may involve TPH1. First, we showed that TPH1 was expressed in rat astrocytes. Next, we determined that OM-LV20 impacted expression changes of TPH1 in CTX-TNA2 cells and exhibited a protective effect on the decrease in cell viability and catalase (CAT) levels induced by hydrogen peroxide. Importantly, we also found that TPH1 expression induced by OM-LV20 may be related to the level of change in the pituitary adenylate cyclase-activating peptide type 1 receptor (PAC1R) and to the JNK signaling pathways, thereby exerting a protective effect on astrocytes against oxidative stress. The protective effects of OM-LV20 likely occur *via* the ‘PAC1R/JNK/TPH1’ axis, thus highlighting TPH1 as a novel antistroke drug target.

Cerebrovascular illnesses, particularly stroke, have gained increasing scientific attention given the world’s aging population (1, 2). Currently, one out of six people will experience a stroke throughout their lifetime, and one out of every 10 patients will die from stroke (3). Stroke treatment alone accounts for 5% to 6% of all medical costs for patients, and the majority will suffer from long-term severe disability, inflicting substantial physical and mental burdens upon them and their families (4, 5). Ischemic stroke (IS) is the most common type of stroke (87% incidence rate) and one of the leading causes of mortality among the elderly (6). Despite the serious consequences of IS, viable therapies remain limited. As a result, the development of effective therapies for IS injury is a major focus of scientific research.

As an important class of neuroglia, astrocytes play a critical role in the central nervous system (CNS) (7). After IS, astrocytes release a variety of nerve factors to resist oxidative stress and inflammatory processes but also shield neurons from injury throughout the IS process (8). Therefore, helping astrocytes survive and sustain normal function is considered one of the most beneficial strategies against cerebral IS injury.

Serotonin (5-HT) is a monoamine neurotransmitter in the brain. It can promote angiogenesis and neurogenesis, inhibit neuroinflammation, and reduce oxidative stress in brain cells and is thus important for normal CNS operation (9). Tryptophan hydroxylase (TPH), a key rate-limiting enzyme for 5-HT synthesis, can be divided into TPH1 and TPH2 subtypes (9). TPH1 is mainly expressed in peripheral tissues expressing 5-HT, such as the skin and intestines, but is rarely expressed in the CNS(10). Recently, however, several studies have reported that TPH1 is correlated with the CNS(11–15). Notably, in our and other previous studies, the level of TPH1 in brain tissue was found to be significantly decreased in rats after stroke (10, 11), suggesting that TPH1 may have a protective effect on nerve damage following stroke.

Due to the novel structure and functional diversity, peptides are widely used to elucidate human disease mechanisms (12, 13). For example, nerve growth factor in snake venom has been used to clarify diseases such as pheochromocytoma (14); captopril, an antihypertensive drug angiotensin-converting enzyme inhibitor, has been applied to elucidate the pathophysiological mechanism of the human cardiovascular system; and hypoglycemic agent insulin has played an important role in diabetes (12, 15). Thus, as molecular probes, peptides are closely related to the analysis of human diseases.

We previously identified a small molecule peptide, named OM-LV20, which showed significant neuroprotective effects in rats following IS, with its underlying mechanism involving the regulation of the mitogen-activated protein kinase (MAPKs) and BDNF/Akt signaling pathways as well as TPH1, pituitary adenylate cyclase-activating peptide type 1 receptor (PCA1R), and cAMP levels (11). Here, to explore the protective mechanisms at the cellular level, we studied the effects of OM-LV20 on astrocytes under oxidative stress. Interestingly, TPH1 was expressed in the rat astrocytes, and OM-LV20, as an exogenous molecular probe, played a neuroprotective role *via* the molecular ‘PAC1R/JNK/TPH1’ axis. We investigated the specific protective mechanism of OM-LV20 regarding the improvement of astrocytes after IS and explored TPH1 as a novel neuroprotective target. Thus, this research provides a potential new drug molecule and targeting strategy for IS treatment and prevention.

## Results

### Expression of TPH1 in rat astrocytes

**Figure 1:**
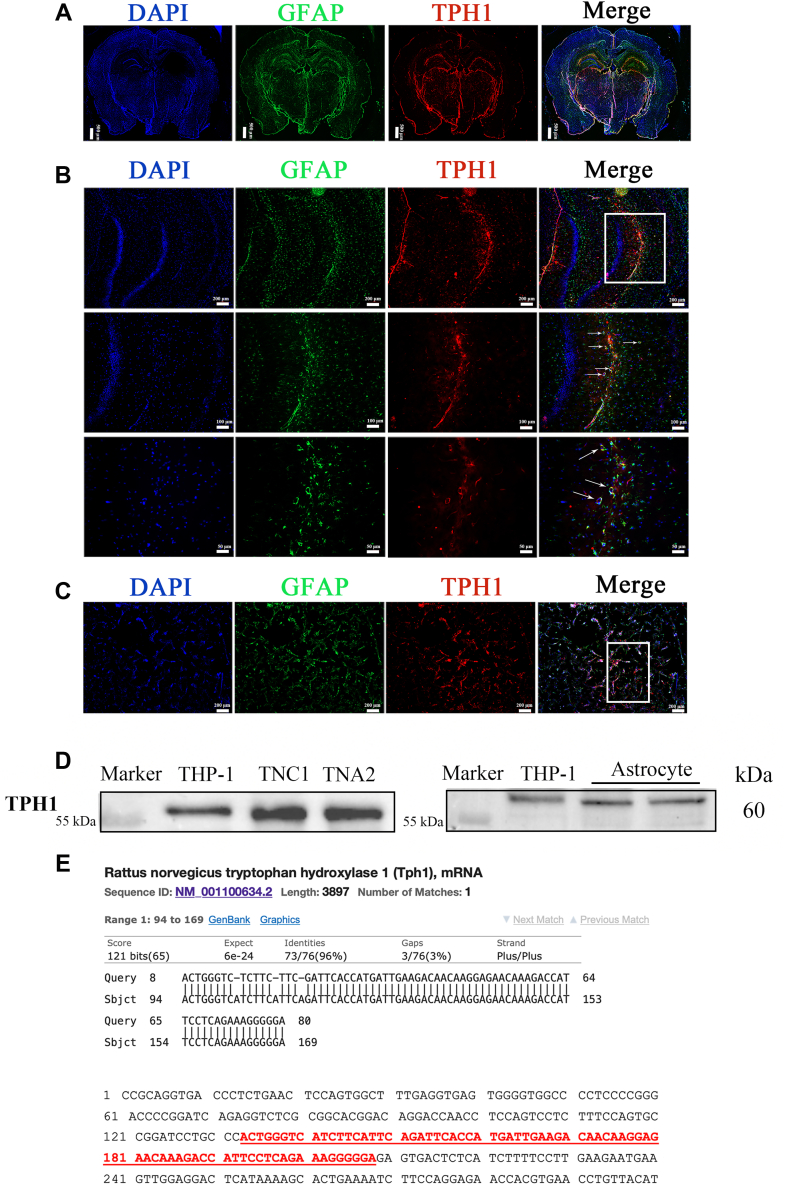
**Expression of TPH1 in rat tissue and astrocytes.***A*, whole brain section showing significant expression of TPH1 in hippocampus and thalamus. *B*, immunofluorescence images showing that TPH1 was successfully colocated with astrocytes in rat hippocampal CA1 region. *C*, immunofluorescence images showing that TPH1 was successfully colocated with rat primary astrocytes. *D*, verification of TPH1 protein expression in primary astrocytes and astrocyte cell lines of rats. THP-1 cells were used as a positive control. n = 3. *White* boxes represent attention-attracting and enlarged sections. *E*, detectable TPH1 mRNA in astrocytes and confirmation of RT-qPCR-amplified product sequence with rat *TPH1* gene sequence. RT-qPCR; quantitative real-time PCR.

We first explored TPH1 expression at the tissue level. As shown in Fig. 1*A*, TPH1 was expressed in the rat brain, with significant distribution in the thalamus, raphe, and CA1 region of the hippocampus. Then, we applied immunofluorescence double labeling to colocalize TPH1, glial fibrillary acidic protein (GFAP) (astrocytes), and 4,6-diamidino-2-phenylindole (DAPI) (nucleus) in the hippocampus and primary astrocytes. TPH1 was successfully colocalized with astrocytes in the hippocampus and primary cells (marked by arrow), demonstrating that TPH1 was expressed in the rat astrocytes (Fig. 1, *B* and *C*).

Secondly, primary astrocytes and two astrocyte cell lines (DI-TNC1 and CTX-TNA2) from rats were used to clarify the expression of TPH1 at the cell level. The expression of TPH1 in the human myeloid leukemia mononuclear cells (THP-1) cells was validated; hence, the THP-1 cells were employed as a positive control. As illustrated in Fig. 1*D*, TPH1 was expressed in the primary astrocytes and astrocyte cell lines. The quantitative real-time PCR (RT-qPCR) amplicon products of the primary astrocytes and DI-TNC1 and CTX-TNA2 cell lines were matched with the rat *TPH1* gene in the NCBI database (sequence ID: NM_001100634.2) (Fig. 1*E*). Thus, these findings indicate that TPH1 is expressed in rat astrocytes.

### Hydrogen peroxide (H_2_O_2_) and OM-LV20 both increase TPH1 levels in CTX-TNA2 cells

**Figure 2:**
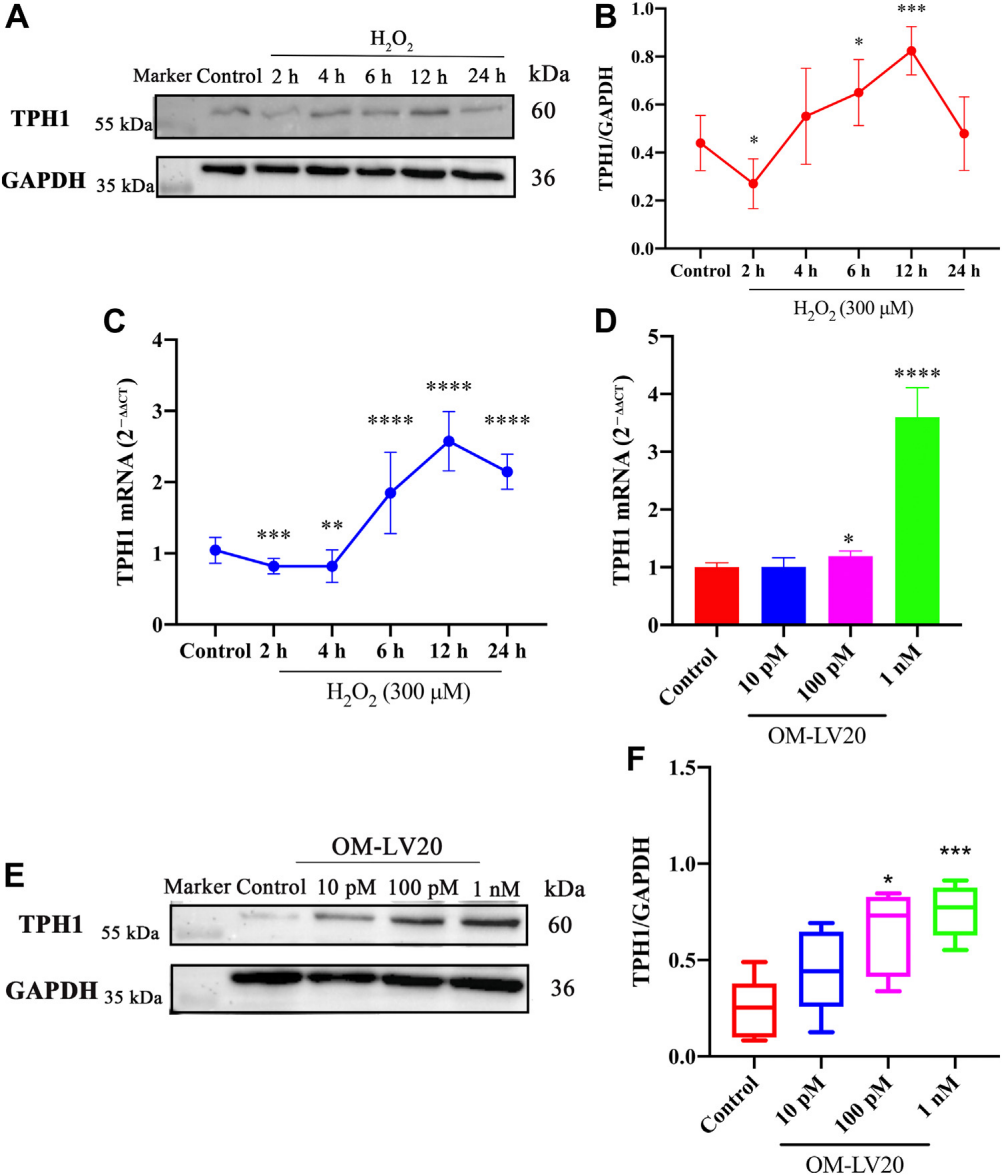
**Promotion effects of OM-LV20 on TPH1.***A*, representative Western blots of TPH1 expression. After H_2_O_2_ stimulation, TPH1 first decreased, then increased. *B*, histogram of protein expression of TPH1. n = 5. *C*, TPH1 mRNA levels after H_2_O_2_ stimulation. n = 5. *D*, TPH1 mRNA levels after OM-LV20 treatment. n = 4. *E*, TPH1 protein representative Western blots after OM-LV20 treatment. *F*, histogram of protein expression of TPH1. n = 5. Data are means ± SEM. ∗*p* < 0.05, ∗∗ *p* < 0.01, ∗∗∗ *p* < 0.001, ∗∗∗∗ *p* < 0.0001.

As shown in Fig. 2, *A* and *B*, TPH1 expression decreased at 2 h after H_2_O_2_ stimulation but then increased at 4, 6, 12, and 24 h, consistent with the RT-qPCR results in Fig. 2*C*. The CTX-TNA2 cells were pretreated with different concentrations of OM-LV20 (10 pM, 100 pM and 1 nM) for 12 h. Results showed that 100 pM and 1 nM OM-LV20 promoted the TPH1 mRNA and protein expression levels (Fig. 2, *D*–*F*).

### OM-LV20 sustains CTX-TNA2 cell viability and improves catalase level induced by H_2_O_2_ treatment

**Figure 3:**
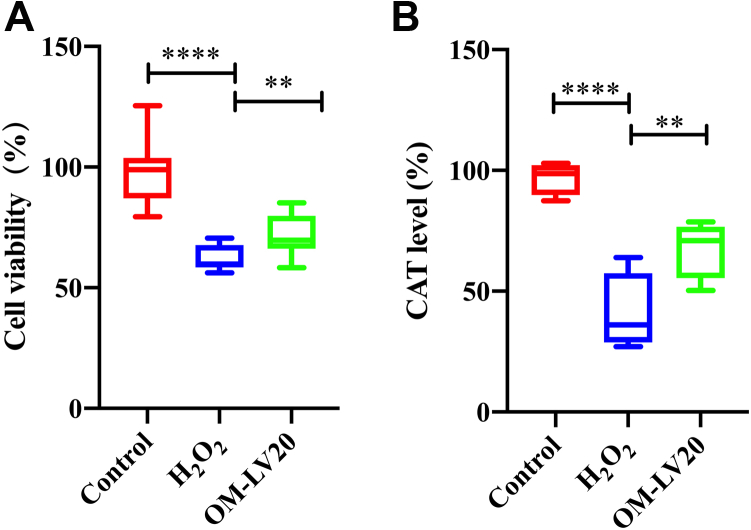
**Effects of OM-LV20 on cell viability and CAT level following H**_**2**_**O**_**2**_**stimulation.***A*, OM-LV20 exhibited a protective activity against H_2_O_2_ stimulation in CTX-TNA2 cells. n = 3. *B*, OM-LV20 improved abnormal decrease in CAT induced by H_2_O_2_ stimulation in CTX-TNA2 cells. Data are means ± SEM. n = 4, ∗∗ *p* < 0.01, ∗∗∗∗ *p* < 0.0001. Control group data were normalized to 100% and other data were normalized accordingly. CAT, catalase.

Before H_2_O_2_ treatment, cells were preadministered with 1 nM OM-LV20 for 12 h (37 °C). As illustrated in Fig. 3*A*, compared with the control group, H_2_O_2_ treatment significantly reduced cell viability by 56.33% ± 14.44%. After OM-LV20 application, cell viability increased by 10.07% ± 8.37%, indicating that OM-LV20 protected cell viability against oxidative stress. H_2_O_2_ stimulation significantly decreased the level of catalase (CAT), whereas OM-LV20 treatment effectively reversed this decrease (increased by 26.92% ± 13.56%) (Fig. 3*B*).

### Changes in MAPK signaling pathway and PAC1R and TPH1 levels following OM-LV20 pretreatment in H_2_O_2_-stimulated CTX-TNA2 cells

**Figure 4:**
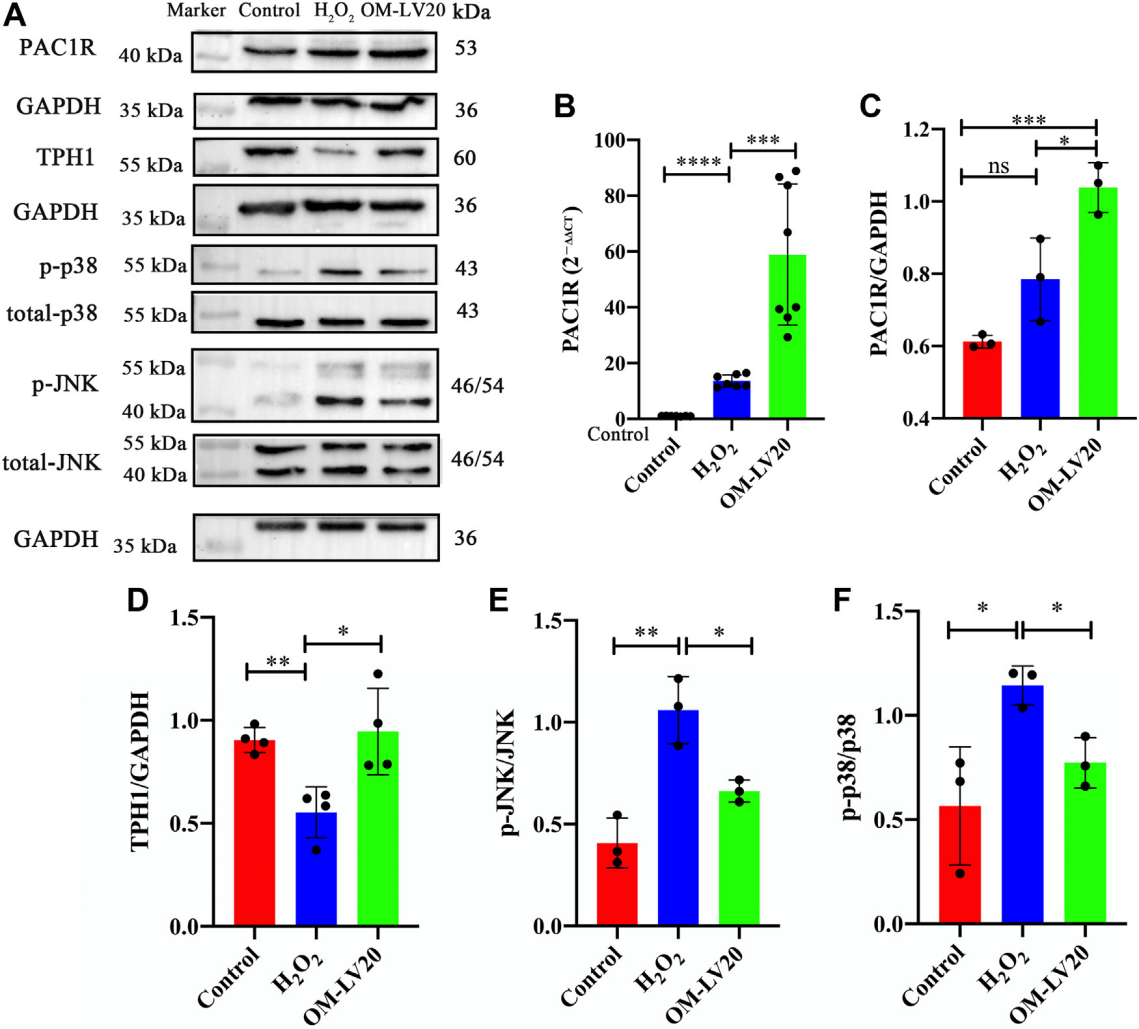
**OM-LV20 inhibited phosphorylation of MAPK signaling pathway and increased levels of TPH1 and PAC1R in CTX-TNA2 cells under H**_**2**_**O**_**2**_**stimulation.***A*, representative Western blots of MAPK, TPH1, and PAC1R protein levels in CTX-TNA2 cells. *B*, OM-LV20 significantly promoted mRNA level of PAC1R. *C*–*F*, histogram of PAC1R, TPH1, p-JNK, and p-p38 protein expression, respectively. Data are means ± SEM. n = 3, ∗*p* < 0.05, ∗∗ *p* < 0.01, ∗∗∗ *p* < 0.001, ∗∗∗∗ *p* < 0.0001.

After H_2_O_2_ stimulation, the mRNA level of PAC1R increased 12.57 ± 2.03-fold compared to that in the control group (Fig. 4*B*). OM-LV20 pretreatment increased the PAC1R mRNA and protein levels by 54.33 ± 28.18-fold and 0.42 ± 0.07-fold, respectively (Fig. 4, *A*–*C*). TPH1 expression was significantly decreased following H_2_O_2_ treatment, whereas OM-LV20 pretreatment alleviated this reduction, resulting in a 0.39 ± 0.15-fold increase (Fig. 4, *A* and *D*). H_2_O_2_ stimulation increased the mRNA level of PAC1R, while OM-LV20 application increased both the mRNA and protein levels of PAC1R. Thus, the peptide increased both PAC1R and TPH1 to resist damage caused by H_2_O_2_.

To investigate the MAPK signaling pathway following H_2_O_2_ treatment, cells were pretreated with OM-LV20 (1 nM) at 37 °C for 12 h before H_2_O_2_ stimulation, with the levels of p-JNK and p-p38 then detected. After H_2_O_2_ treatment, the levels of p-JNK and p-p38 markedly increased (*p* < 0.05 and *p* < 0.001, respectively) but both decreased under OM-LV20 pretreatment (0.40 ± 0.16-fold and 0.37 ± 0.12-fold, respectively (Fig. 4, *A*,*E*, and F).

### OM-LV20 regulated TPH1 by inhibiting p-JNK level in CTX-TNA2 cells

**Figure 5:**
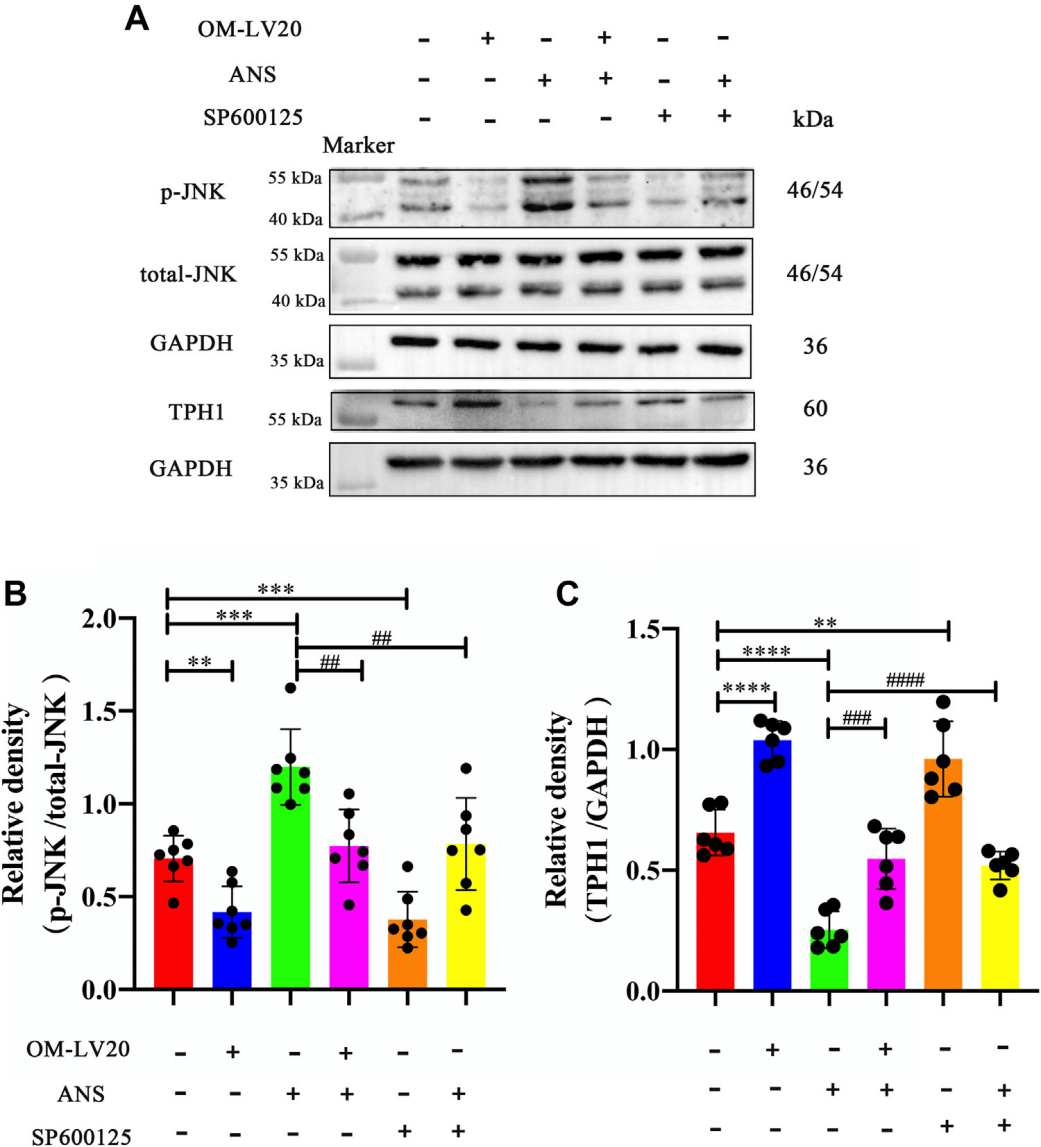
**OM-LV20 regulated TPH1 by inhibiting p-JNK phosphorylation.***A*, representative Western blots of p-JNK and TPH1 protein levels in CTX-TNA2 cells. *B*, histogram of p-JNK/JNK protein expression. n = 7. *C*, histogram of TPH1 protein expression. n = 6. ANS is a potent JNK activator and SP600125 is a highly selective JNK inhibitor. Data are means ± SEM. ∗∗ *p* < 0.01, ∗∗∗ *p* < 0.001, ∗∗∗∗ *p* < 0.0001; ## *p* < 0.01, ### *p* < 0.001, #### *p* < 0.0001. ANS, anisomycin.

As shown in Fig. 4, the application of OM-LV20 reduced the level of p-JNK but increased the TPH1 level at the same time. So, to study the relevance between p-JNK and TPH1 levels regulated by OM-LV20, an inhibitor (SP600125) and an activator (anisomycin [ANS]) of the JNK signaling pathway were used. Compared with the control group, administration of ANS increased the p-JNK level; however, in the OM-LV20 + ANS group, p-JNK expression decreased compared with that in the ANS group. Thus, OM-LV20 likely played a role in the decline of p-JNK (Fig. 5, *A* and *B*). Furthermore, simultaneous application of OM-LV20 and ANS decreased TPH1 in the CTX-TNA2 cells (Fig. 5, *A* and *C*), illustrating that the increase in p-JNK expression may partly weaken the promotion of TPH1 by OM-LV20. Comparing the ANS and SP60025 + ANS groups, we found that SP600125 also reversed the increase in p-JNK and decrease in TPH1 caused by ANS, indicating that the level of p-JNK had an effect on TPH1 expression (Fig. 5). Based on the aforementioned results, the level of p-JNK was negatively correlated with the level of TPH1 in CTX-TNA2 cells, and thus, OM-LV20 likely regulates TPH1 by inhibiting the level of p-JNK.

### OM-LV20 regulated TPH1 through PAC1R in CTX-TNA2 cells

**Figure 6:**
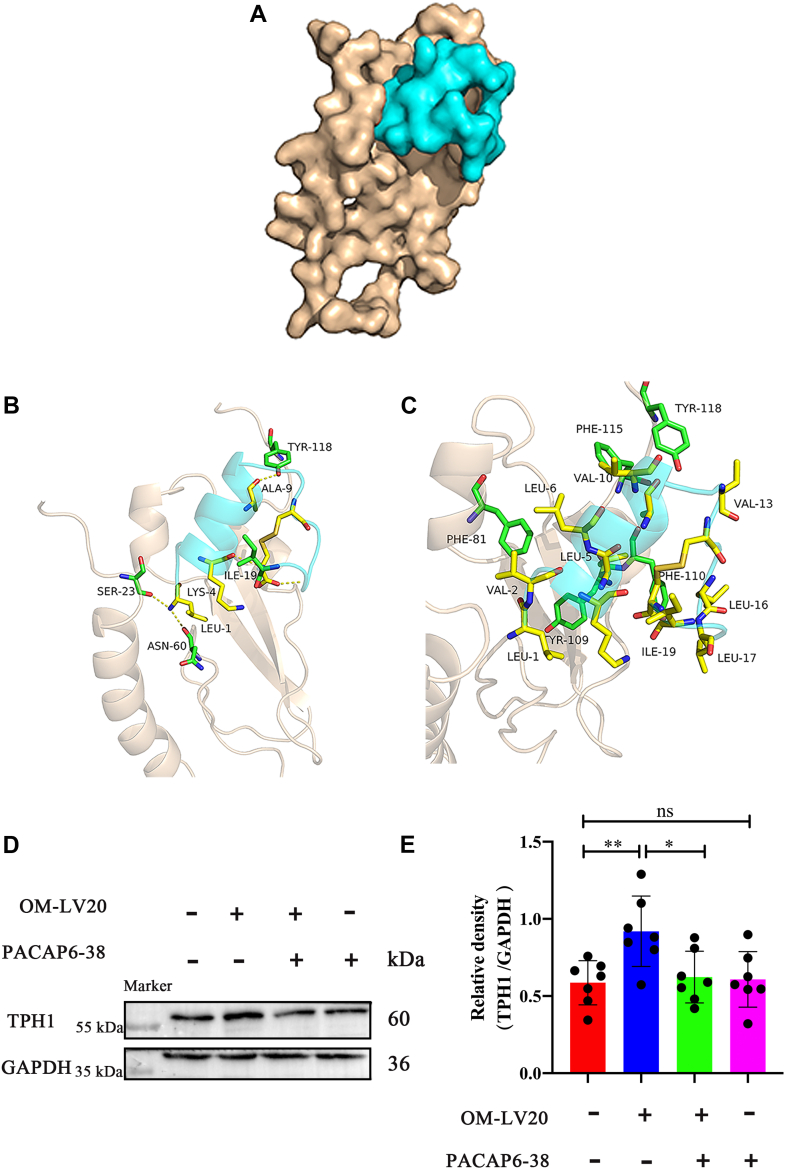
**OM-LV20 regulated TPH1 by binding to PAC1R in CTX-TNA2 cells.***A*, *yellow* represents PAC1R conformation, and *blue* represents OM-LV20 conformation. PAC1R and OM-LV20 showed good shape complementarity. *B*, *yellow* is residue of OM-LV20, and *blue* is PAC1R protein residue. LEU1 formed hydrogen bonds with receptor proteins SER23 and ASN60; LYS4 formed hydrogen bonds with receptor protein ILE19; ALA9 formed hydrogen bonds with TYR118. *C*, *yellow* is hydrophobic residue of OM-LV20, and *blue* is hydrophobic residue of PAC1R protein. They showed hydrophobic accumulation and Van der Waals interactions. *D*, application of PACAP6-38 partly weakened increase in TPH1 by OM-LV20. *E*, histogram of TPH1 protein expression. Data are means ± SEM. n = 7, ∗*p* < 0.05, ∗∗ *p* < 0.01.

In view of the increase in TPH1 levels in cells and tissues following OM-LV20 administration, molecular docking simulation was used to explore the possible relationship between OM-LV20 and PAC1R. Results showed that OM-LV20 and PAC1R had a good shape complementarity (Fig. 6*A*). As seen in Fig. 6*B*, OM-LV20 likely forms hydrogen bonds with PAC1R through TYR-118, ALA-9, ILE-19, LYS-4, LEU-1, ASN-60, and SER-23. Furthermore, as seen in Fig. 6*C*, the peptide likely shows hydrophobic accumulation and Van der Waals interactions with PAC1R through TYR-118, PHE-115, VAL-13, VAL-10, LEU-6, LEU-5, PHE-81, PHE-110, LEU-16, LEU-17, ILE-19, VAL-2, LEU-1, and TYR-109. These are crucial to the binding stability of OM-LV20 with PAC1R.

By using a PAC1R antagonist (PACAP6-38), we also investigated whether OM-LV20 influenced TPH1 through PAC1R. Results demonstrated that TPH1 expression increased 0.33 ± 0.13-fold after OM-LV20 treatment, whereas concurrent treatment with OM-LV20 and PACAP6-38 partly reversed this increase (Fig. 6, *D* and *E*). These results confirm that OM-LV20 may regulate TPH1 by binding to PAC1R.

### Overexpression of TPH1 did not protect CTX-TNA2 cell viability from H_2_O_2_ stimulation but increased the CAT level

**Figure 7:**
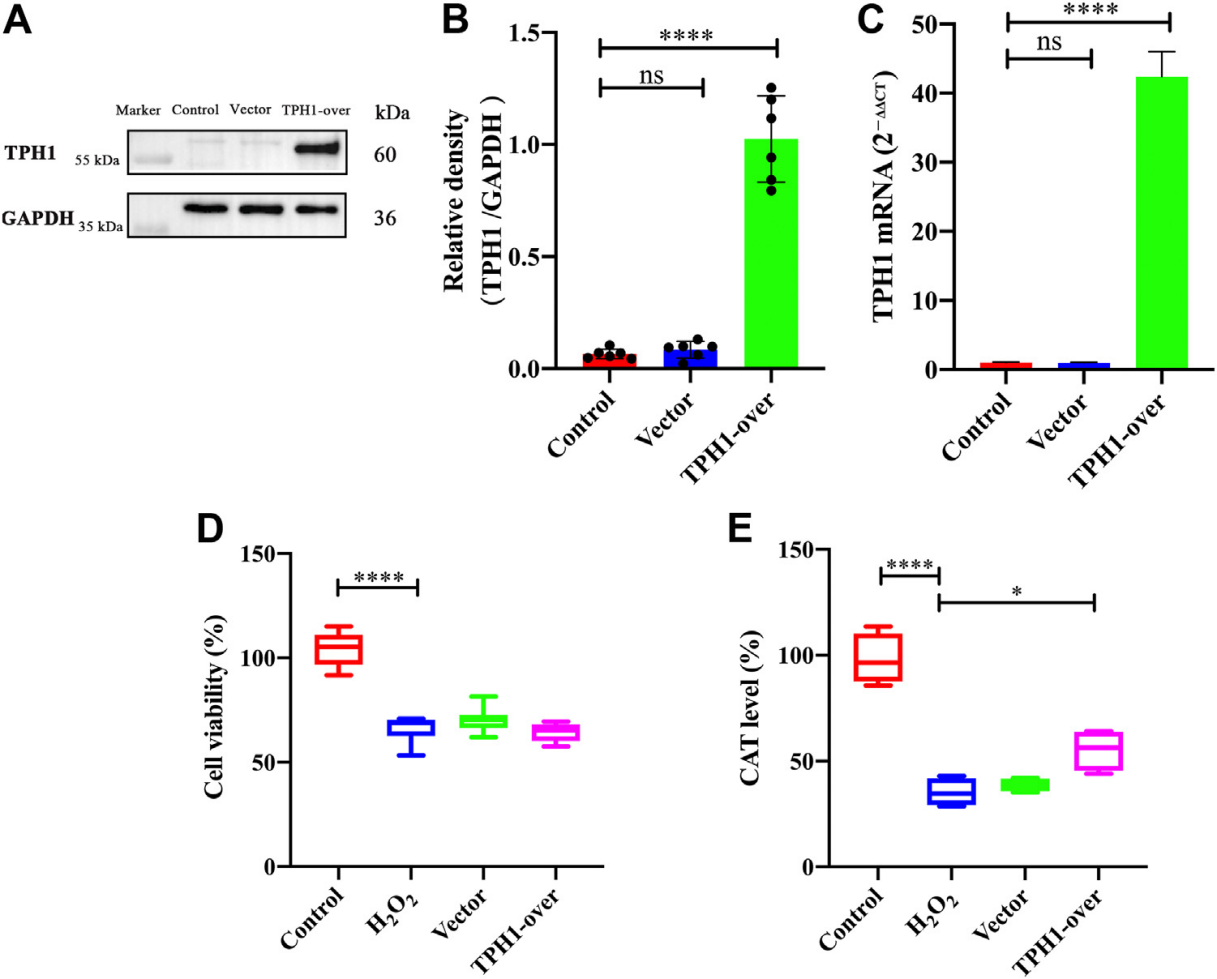
**Effects of TPH1 overexpression on cell viability and CTA level in CTX-TNA2 cells stimulated by H**_**2**_**O**_**2**_**.***A*, TPH1 was successfully overexpressed in CTX-TNA2 cells. *B*, histogram of TPH1 protein expression. n = 6. *C*, verification of mRNA level with TPH1 overexpression. n = 4. *D*, overexpression of TPH1 had no protective effects on CTX-TNA2 cell viability stimulated by H_2_O_2_. n = 3. *E*, TPH1 overexpression reversed abnormal CAT decrease induced by H_2_O_2_. n = 4. Vector group on behalf of empty virus vector. Data are means ± SEM. ∗*p* < 0.05, ∗∗∗∗ *p* < 0.0001. Control group data were normalized to 100% and other data were normalized accordingly. CAT, catalase.

After TPH1 overexpression, RT-qPCR and Western blot analyses were performed to detect TPH1 expression in the CTX-TNA2 cells. Compared with the control group, the mRNA and protein levels of TPH1 increased 41.4 ± 3.0-fold and 0.96 ± 0.17-fold, respectively (Fig. 7, *A*–*C*). No significant differences were observed between the vector (negative control) and control groups, demonstrating that TPH1 was effectively overexpressed in the cells, which were used in subsequent experiments.

Treatment with H_2_O_2_ led to a decrease in cell viability and CAT level. As shown in Fig. 7*D*, overexpression of TPH1 had no protective effect on cell viability damage. As a classic antioxidant factor that maintains homeostasis of the oxidative system in cells and the body, a decrease in CAT indicates that oxidative stress has caused the body system to enter an imbalanced state (16). As seen in Fig. 7*E*, CAT level decreased by 62.79% ± 8.21% after H_2_O_2_ treatment but increased by 19.92% ± 4.11% after TPH1-overexpression.

## Discussion

IS is one of the main causes of disability worldwide, and its incidence increases with age (17). Despite considerable effort, prevention and therapy remain ineffective and a serious clinical challenge (17, 18). Therefore, the identification of new targets and the development of novel candidate drugs for effective IS treatment is critical.

In our previous study on the protective effects of OM-LV20 on cerebral ischemia-reperfusion (I/R) injury in rats, TPH1 was significantly upregulated following OM-LV20 treatment (compared with the model group) and significantly decreased after cerebral I/R (11). Thus, the application of OM-LV20 not only had a protective effect on brain injury but also improved TPH1 levels (11). According to these results, TPH1 may be a therapeutic target for nerve injury caused by cerebral I/R.

In the normal operation of the CNS, TPH1 participates in a variety of mental and neurobehavioral-related processes. For example, genetic studies have shown that some psychiatric diagnostic categories, such as TPH1, are related to serotonin function (19). Other research has reported that TPH1 is associated with schizophrenia (20). Furthermore, in mouse models of reserpine-induced depression, scopolamine can reverse the decrease in TPH1 levels in the hippocampus and prefrontal cortex (21). In rats, the mRNA level of TPH1 decreases in the brain after stroke but acupuncture intervention significantly increases TPH1 levels (10). Although TPH1 was primarily thought to be distributed in peripheral tissues and not expressed in the brain, more recent studies have suggested that it is expressed in several brain regions, such as the hippocampus, thalamus, and amygdala (21). In addition, TPH1 appears to be pre-expressed in later stages of brain development (22, 23). However, whether TPH1 is definitively expressed in the brain and at which specific sites remains controversial.

To clarify the aforementioned question, we explored TPH1-specific expression in the rat brain. Results showed that TPH1 was expressed in the brain, mainly in the hippocampal CA1 region, thalamic region, and cerebral raphe (Fig. 1*A*). The hippocampus is one of the most important structures in the brain and is closely related to cognition, learning, and memory (24). The CA1 area is located in the vulnerable ‘head’ area of the hippocampus and is thus highly sensitive to hypoxia, lesions, and other injuries (25, 26). Combined with the significant expression of TPH1 in the CA1 region, we speculated that TPH1 may play a role in neuroprotection, especially in maintaining hippocampal homeostasis.

Astrocytes account for a large proportion of glial cells in the hippocampus (27). As astrocytes are the most abundant cell type in the CNS and can affect neuronal survival under physiological and pathological conditions, astrocyte-related treatment strategies are an important direction in IS treatment (7, 8, 28). Astrocytes are also reported to have a variety of neurotransmitter receptors, such as dopamine, acetylcholine, and serotonergic receptors (29). Selective serotonin reuptake inhibitors, which are commonly used as antidepressants, can play a neuroprotective role by activating 5-HT2B receptors in astrocytes and neurons (9). Moreover, tryptophan, the precursor for 5-HT synthesis, can be decomposed by indoleamine 2,3-dioxygenase to form canine urinary tryptophan, with indoleamine 2,3-dioxygenase not only found in tissues outside the liver but also in brain endothelial cells, macrophages, and astrocytes (29). Thus, we speculated that TPH1 is likely to be expressed in astrocytes, as confirmed in the current study. Notably, TPH1 was expressed in hippocampal astrocytes, primary hippocampal astrocytes, and two rat astrocyte cell lines. In addition, we compared the RT-qPCR-amplified sequence with the rat *TPH1* gene sequence for confirmation (Fig. 1). These findings provided strong evidence for the existence of TPH1 in rat astrocytes (Fig. 1). Therefore, we focused on the neuroprotective effects of TPH1 on astrocytes.

The pathological process of cerebral IS is complex, and its occurrence and development involve a variety of mechanisms. Among these mechanisms, oxidative stress is one of the main causes of IS injury (30, 31). When IS occurs, cells are in a state of ischemia and hypoxia, which leads to the consumption of antioxidants (such as SOD and CAT), disruption of antioxidant balance, destruction of the function and survival of nerve cells, and aggravation of IS (32, 33). Thus, in the current study, we treated astrocytes with H_2_O_2_ to simulate oxidative stress *in vitro*. We found that the application of OM-LV20 not only promoted TPH1 levels in brain tissue (11) but also increased the TPH1 level in astrocytes in a concentration-dependent manner (Fig. 2). In addition, OM-LV20 significantly improved the decrease in cell viability and CAT level induced by H_2_O_2_ (Fig. 3). Based on further analysis, the protective effects of OM-LV20 on rat astrocytes under oxidative stress may be through inhibiting the phosphorylation of the JNK signaling pathway and increasing TPH1 and PAC1R levels (Fig. 4). Under H_2_O_2_ stimulation, the level of TPH1 in the astrocytes decreased significantly and then increased to a peak at 12 h. The reason for this may be that oxidative stress first consumes intracellular TPH1, after which the cell increases TPH1 due to cell homeostasis imbalance. We also observed that OM-LV20 increased TPH1 levels in the cells after 12 h of treatment. We further explored the function of TPH1 by constructing a stable TPH1-overexpression CTX-TNA2 cell line. Under oxidative stress, the overexpression of TPH1 did not protect cell viability but increased the level of CAT (Fig. 7). Of note, Fig. 2 and 3 demonstrate that while overexpression of TPH1 in astrocytes alone did not ameliorate the H_2_O_2_-induced decrease in cell viability, it increased CAT levels. Furthermore, OM-LV20 increased TPH1 levels, improved cell viability reduction, and increased CAT levels. This may be partly attributable to the protective effects of OM-LV20, including activation of the MAPK signaling pathway and increased PAC1R and TPH1 levels (Fig. 4). OM-LV20 also had a greater impact on the increase in CAT under oxidative stress compared to the TPH1 overexpression group (Figs. 3 and 7). Therefore, the protective effects of the peptide, particularly against oxidative stress, may be complex and include more than increasing TPH1 levels. In addition, overexpression of TPH1 may protect cells from harm caused by oxidative stress primarily by increasing CAT levels.

Combined with the previous study showing that TPH1 overexpression improves renal interstitial fibrosis and reduces inflammation and oxidative stress in renal I/R mice (34), indicating a close relationship between TPH1 level and oxidative stress response, our findings suggest that TPH1 may play a neuroprotective role by balancing the redox response.

Interestingly, using inhibitors and activators of the JNK signaling pathway, we found that OM-LV20 partly regulated TPH1 through p-JNK expression (Fig. 5). Various studies have shown that the MAPK signaling pathway is correlated with TPH1 expression. For example, deletion of 5-HT in TPH1 KO mice increases the phosphorylation of p-JNK, while supplementation with 5-HT reduces JNK activation (35). In enterochromaffin cell hypoxia models, the MAPK signaling pathway regulates TPH1 expression, with TPH1 phosphorylation correlated with MAPK phosphorylation (36). In MDA-MB-231 cells, 5-HT treatment regulates the expression of the target protein CTSS by activating ERK and p38, while CTSS expression decreases significantly after TPH1 KO (37). Therefore, based on our results and those of others, there appears to be an interaction between the MAPK signaling pathway and TPH1 levels. Activation of the MAPK signaling pathway regulates the expression of TPH1, and TPH1 regulates the phosphorylation of the MAPK signaling pathway. Therefore, OM-LV20 may affect the level of TPH1 by regulating the MAPK signaling pathway and thereby assist cells in resisting oxidative stress.

**Figure 8:**
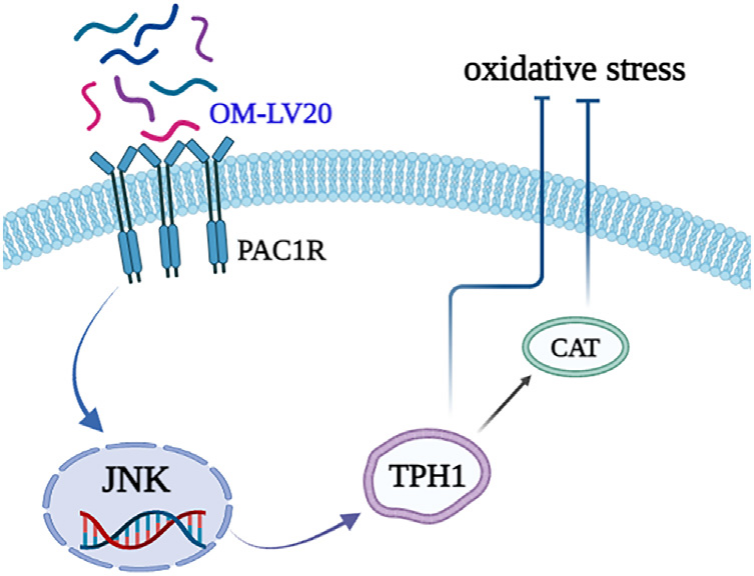
**Neuroprotective mechanism of OM-LV20 in astrocytes.** Peptide OM-LV20 exerts protective effects on astrocytes *via* the ‘PAC1R/JNK/TPH1’ axis to resist oxidative stress.

In our previous study, the application of OM-LV20 significantly upregulated the level of PAC1R in rat brains and astrocytes (11). PAC1R is a specific receptor with high affinity for the neurotransmitter PACAP, and the neuroprotective effects of PACAP depend on the activation of PAC1R (38). Thus, we explored whether OM-LV20 plays a neuroprotective role by regulating the downstream signaling pathway and binding to PAC1R on the cell membrane. Firstly, OM-LV20 and PAC1R showed docking potential (Fig. 6*A*). Using PACAP6-38 (antagonist of PAC1R) indicated that OM-LV20 may regulate TPH1 levels by combining with PAC1R (Fig. 6). Several studies have revealed a potential regulatory relationship between PAC1R and the MAPK signaling pathway, especially the JNK pathway. For example, intravenous application of the PAC1R agonist maxadilan can activate astrocytes in the spinal cord and increase the phosphorylation of JNK and ERK, while application of a PAC1R inhibitor can inhibit p-ERK levels in astrocytes (39). In human corneal endothelial cells, PACAP can specifically bind to PAC1R to activate the ERK1/2 signaling pathway to maintain cell survival, proliferation, and corneal endothelial integrity (40). Moreover, the application of PACAP6-38 in human trophoblast cells can promote the phosphorylation of ERK1/2 and JNK and inhibit the phosphorylation of p38 (41). Hippocampal pyramidal cells and astrocytes can increase the expression of PAC1R after rat I/R. Reactive astrocytes can release interleukin 6 (IL-6) and increase the expression of PAC1R, with PACAP, PAC1R, and IL-6 cooperatively inhibiting the death of hippocampal apoptotic cells. This mechanism is partly achieved by inhibiting the increase in JNK and p38 activities. This suggests that increasing PAC1R may affect on the inhibition of the JNK signaling pathway in astrocytes (42, 43). As such, we inferred that the neuroprotective effects of OM-LV20 may occur *via* the ‘PAC1R/JNK/TPH1’ axis (Fig. 8).

In summary, this study is the first to report on the expression of TPH1 in rat astrocytes. We also determined that the neuroprotective effects of OM-LV20 on rat astrocytes under oxidative stress may occur *via* the ‘PAC1R/JNK/TPH1’ molecular axis, suggesting the possibility of TPH1 as a neuroprotective target. Thus, this study provides potential antistroke drug molecules and new targeting strategies for the treatment and prevention of nervous system diseases, especially IS.

## Experimental procedures

### Animals

Adult Sprague–Dawley (SD) rats (280–300 g) and neonatal SD rats (24–48 h) were obtained from the Laboratory Animal Department of Kunming Medical University. The rats were housed under a constant temperature and had unlimited access to food and water. All animals were treated in strict accordance with the Chinese Medical Laboratory Animal Management Regulations. Animal care and treatment were approved by the Ethics Committee of Kunming Medical University (License number: KMMU2020077) and carried out in accordance with the regulations and requirements.

### Peptide synthesis

The peptide OM-LV20 (LVGKLLKGAVGDVCGLLPIC) was produced by Wuhan Bioyeargene Biotechnology Co, Ltd *via* solid-phase synthesis with a purity >95%.

### Culture and purification of primary rat astrocytes

Newborn (24–48 h) SD rats were selected, and their brains were removed in an aseptic environment. After carefully dissecting the remaining meninges, blood vessels, and bleeding points under a vertical microscope, only the hippocampus was preserved. Brain tissue was digested with 0.25% trypsin and incubated in a 37 °C incubator for 5 to 15 min until there was no obvious tissue mass. Trypsin digestion was then terminated in Dulbecco’s modified Eagle’s medium/F12 medium (DMEM/F12, BI) containing 10% fetal bovine serum (FBS, BI). The obtained liquid was filtered through a 75 μm cell sieve and then centrifuged at 1000×*g* at room temperature (RT) for 5 min. The precipitated cells were resuspended at 2 × 10^6^ in 75 cm^2^ culture bottles, then inoculated in a 37 °C incubator. After 24 h, the FBS concentration in the cell culture medium was changed from 10% to 20%, with another 7 days of culture at 37 °C.

After 8 days of culture, the cell medium was changed, followed by shaking for 16 to 18 h at 200×*g* and 37 °C. After shaking, the scattered cells suspended in the culture medium were discarded. The adherent cells were washed with PBS 3 times, then digested with 0.25% trypsin, and centrifuged at 1000×*g* for 5 min at RT. The precipitated cells were collected and inoculated in a 75 cm^2^ culture flask. After 30 min, the supernatant was transferred to a new culture flask or well plate. Floating cells were discarded after 1 h, and the adherent cells were used for generation, identification, and experimentation.

### Cell culture and H_2_O_2_ stimulation

The THP-1, DI-TNC1, and CTX-TNA2 cell lines were all cultured in DMEM/high glucose medium (BI) supplemented with 1% antibiotics (100 unit/ml penicillin, 100 unit/ml streptomycin) and 10% FBS at 37 °C in a humidified incubator with 5% CO_2_. Cells were seeded in 6-well plates (1.2 × 10^6^ cells/well) and 96-well plates (4 × 10^3^ cells/well). After cells reached 80% to 90% confluency, the medium was removed and the cells were washed with PBS 3 times, after which 300 μM H_2_O_2_ was added to the wells for 2, 4, 6, 12, and 24 h of stimulation, respectively.

### RT-qPCR assay

Total RNA from the CTX-TNA2 cell line was extracted using a Total RNA Extraction Kit (Tiangen Biotech). RNA (1.5 μg) was then reverse transcribed into complementary DNA using a Prime Script Reagent Kit (GeneCopoeia). RT-qPCR was performed using the following primers: PAC1R (5′-ACTACCTGTCGGTGAAGGCTCTC-3′ and 5′-CGGAAGCGGCACAGGATGACC-3′), TPH1(5′-GGCTTTGAGGTCCTCTTTCCA-3′ and 5′-CCCCCTTTCTGAGGAATGGTC-3′), and ACTIN (5′-CAGCCTTCCTTCCTGGGTATG-3′ and 5′-TAGAGCCACCAATCCACACAG-3′) from Sangon Biotech.

### Double immunofluorescence assay

Rats were anesthetized with 6% pentobarbital sodium and placed on an icebox, then perfused with 0.9% normal saline and 4% paraformaldehyde. After the brain was removed, the tissue was placed in 4% paraformaldehyde for 24 to 36 h and then dehydrated with sucrose under a concentration gradient of 15%, 20%, and 30%. After the brain sank, optimal cutting temperature compound was used for freeze embedding at −80 °C. The frozen brain tissue was cut into 10 μm sections along the coronal plane. These sections were then placed in PBS for 10 min hydration and used for subsequent immunofluorescence staining experiments. The primary astrocytes were inoculated in 24-well plates containing climbing slides. After 24 h, the medium in the plates was discarded and the cells were washed with PBS. All slices and cells were incubated with 0.3% Triton-X-100 for 20 min and then blocked with 10% sheep serum at RT for 1 h. The serum was removed, and the cells and slices were then incubated with primary antibodies TPH1 (1:50; Invitrogen) and GFAP (1:200; Cell Signaling Technology) at 4 °C overnight. On the second day, the slices and cells were balanced to RT for 30 min, washed with PBS with 0.1% 23 times (5 min each), incubated with secondary antibodies FITC and Cy3 (1:200; Proteintech) at RT for 1 h, then finally sealed with 4,6-diamidino-2-phenylindole solution in the dark and placed in a wet box for photography.

### Lentiviral infection

The lentiviral vector for TPH1 overexpression (Gene ID: NM_001100634.2) and lentiviral empty vector (Vector) were provided by Hanheng Biotechnology Co, Ltd (Shanghai, China). For overexpression, the CTX-TNA2 cells were seeded at 3 × 10^5^ cells/well into 6-well plates. After cells adhered to the walls, they were gently washed with PBS 3 times. The virus was diluted with complete medium and added to the plate with a multiplicity of infection of 30. After 24 h of infection, the entire solution was changed with whole cell culture medium. Enhanced GFP expression was observed using a fluorescence microscope for 48 to 72 h. Puromycin (4 μg/ml; Solarbio) was screened 3 days after virus infection to obtain successfully transfected cells. The stable cell line was selected and cultured with 2 μg/ml puromycin for the following experiments.

### Cell viability assay

Cells were seeded into 96-well plates at 4 × 10^3^ cells/well. After adhering to the walls, cells were replaced by serum-free medium and divided into five groups, that is, control, H_2_O_2_, OM-LV20 (1 nM), vector, and TPH1-overexpressed groups. H_2_O_2_ stimulation (2 h) was performed 12 h after pretreatment with the peptide. Then, 20 μl of 3-(4,5-dimethyl-2-thiazolyl)-2,5-diphenyl-2-H-tetrazolium bromide (MTS, Promega) was added to each well, followed by incubation in a dark incubator for 2 to 4 h at 37 °C. Absorbance was then detected at 490 nm.

### Western blotting assay

CTX-TNA2 cells were seeded into 6-well plates (1.2 × 10^6^ cells/well) and divided into groups. After incubation with OM-LV20 for 12 h or stimulation with H_2_O_2_ (300 μM) for 2 h, the cells were collected for protein extraction and BCA quantitative tests to detect the levels of TPH1, p-JNK, JNK, p-p38, p38, TPH1, and PAC1R.

Cells were seeded into 6-well plates as before. The concentrations of ANS, OM-LV20, inhibitor SP600125, and PACAP6-38 were 5 μM, 1 nM, 10 μM, and 2 μM, respectively. Cells were incubated at 37 °C with drugs for 12 h and stimulated with H_2_O_2_ for 2 h. In the PACAP6-38 + OM-LV20 group, the cells were treated with PACAP6-38 for 2 h and then OM-LV20 was added for another 24 h of incubation. The cells were then collected for protein extraction and bicinchoninic acid assay (BCA) quantitative tests to detect the levels of p-JNK, JNK, and TPH1.

Proteins were separated using 10% to 12% SDS-PAGE. The Western blotting and BCA protocols are discussed in previous research (11). Primary antibodies included the following: GAPDH (1:3000; Proteintech); p-JNK, JNK, p-p38, and p38 (1:2000; Cell Signaling); TPH1 (1:500; Abcam); and PAC1R (1:500; ABclonal). The secondary antibody was purchased from Proteintech (1:5000).

### Docking simulation of peptide OM-LV20 with PAC1R

Pepfold3 was used to construct the initial model of the OM-LV20 peptide (https://bioserv.rpbs.univ-paris-diderot.fr/services/PEP-FOLD3/). The three-dimensional structure of the peptide was constructed and downloaded from the RCSB database (Protein Data Bank ID: 7jqd). Peptide structures were processed using the AutoDock Tool (AutoDock Vina v1.1.2), atomic types were assigned, and partial charges were calculated. During docking, both peptides and proteins remained rigid. The peptide and PAC1R were then connected. All docking parameters were default, and Top1 docking conformation was selected for subsequent molecular dynamics simulation carried out at 50 ns. The binding free energy of the docking compound was calculated using a kinetic trajectory of 25 to 50 ns.

### Measurement of CAT level

Cells were divided into five groups: control, H_2_O_2_, OM-LV20 (1 nM), vector, and overexpressed-TPH1 groups. After the adherent cells were stimulated with H_2_O_2_ (2 h), the culture medium in the well plate was removed. Washed cells with precooled PBS 3 times and 200 μL of lysis buffer (mixed with precooled radioimmunoprecipitation assay and PMSF) (Meilun Biotechnology) was added per well. Cells were centrifuged at 12,000×*g* for 20 min (4 °C) to obtain the supernatant. The CAT level was measured according to the instructions provided with the Nanjing Jiancheng Kit. Finally, absorbance was measured at 405 nm.

### Statistical analysis

Data are shown as means ± SEM. Results were analyzed using GraphPad Prism v8.0 (GraphPad Software Inc). The *t* test or nonparametric Mann–Whitney test was used to determine the statistical significance of differences between two groups. Significance was established at a *p*-value of 0.05 or less.

## Data availability

Data that support the findings of this study are available from the corresponding author upon reasonable request.

## Conflicts of interest

The authors declare that they have no conflicts of interest with the contents of this article.
